# High DNMT1 Expression in Stromal Fibroblasts Promotes Angiogenesis and Unfavorable Outcome in Locally Advanced Breast Cancer Patients

**DOI:** 10.3389/fonc.2022.877219

**Published:** 2022-06-02

**Authors:** Layla A. Al-Kharashi, Asma Tulbah, Maria Arafah, Abdelmonneim M. Eldali, Taher Al-Tweigeri, Abdelilah Aboussekhra

**Affiliations:** ^1^ Department of Molecular Oncology, King Faisal Specialist Hospital and Research Center, Riyadh, Saudi Arabia; ^2^ Department of Pharmacology and Toxicology, Faculty of Pharmacy, King Saud University, Riyadh, Saudi Arabia; ^3^ Department of Pathology, King Faisal Specialist Hospital and Research Center, Riyadh, Saudi Arabia; ^4^ Department of Pathology, King Saud University, Riyadh, Saudi Arabia; ^5^ Department of Biostatistics, Epidemiology and Scientific Computing, King Faisal Specialist Hospital and Research Center, Riyadh, Saudi Arabia; ^6^ Department of Oncology, King Faisal Specialist Hospital and Research Center, Riyadh, Saudi Arabia

**Keywords:** DNMT-1, breast cancer, cancer-associated fibroblasts, VEGF-A, prognosis

## Abstract

**Background:**

Active breast cancer-associated fibroblasts (CAFs) play a leading role in breast carcinogenesis through promoting angiogenesis and resistance to therapy. Consequently, these active stromal cells have significant influence on patient outcome. Therefore, we explored here the role of the DNA methyltransferase 1 (DNMT1) protein in CAF-dependent promotion of angiogenesis as well as the prognostic power of DNMT1 level in both cancer cells and their adjacent CAFs in locally advanced breast cancer patients.

**Methods:**

We applied immunohistochemistry to evaluate the level of DNMT1 in breast cancer tissues and their adjacent normal counterparts. Quantitative RT-PCR and immunoblotting were performed to investigate the role of DNMT1 in regulating the expression of pro-angiogenic genes in active CAFs and also their response to the DNMT1 inhibitors decitabine (DAC) as well as eugenol.

**Results:**

We have shown that DNMT1 controls the pro-angiogenic potential of CAFs both *in vitro* and *in vivo* through positive regulation of the expression/secretion of 2 important pro-angiogenic factors VEGF-A and IL-8 as well as their upstream effectors mTOR and HIF-1α. To confirm this, we have shown that these DNMT1-related pro-angiogenic effects were suppressed by 2 DNMT1 inhibitors decitabine and eugenol. Interestingly, in a cohort of 100 tumors from locally advanced breast cancer patients (LABC), we have shown that high expression of DNMT1 in tumor cells and their adjacent stromal fibroblasts is correlated with poor survival of these patients.

**Conclusion:**

DNMT1 upregulation in breast stromal fibroblasts promotes angiogenesis *via* IL-8/VEGF-A upregulation, and correlates well with poor survival of LABC patients.

## Introduction

Breast cancer (BC) is a high-burden malignancy and a serious threat for women’s health world-wide (1). Despite important advances in BC biology and therapeutic approaches, locally advanced breast cancer (LABC) remains a major clinical issue with unfavorable prognosis and increased risk of locoregional recurrence as well as distant metastasis (2). Unfortunately, the use of neoadjuvant chemotherapy, the standard management therapy for LABC, allows disappearance of the tumor (pathological complete response: PCR) in only 20–30% of cases (3, 4). Therefore, efforts have been made to identify molecular biomarkers and therapeutic targets to predict and improve the response to the various neoadjuvant therapeutic protocols.

Tumor cells reside in a highly complex and heterogeneous tumor microenvironment (TME), which contains several types of cells with various functions and tumorigenic capacities. Among the components of the TME, cancer-associated fibroblasts (CAFs) play important roles in tumor onset and spread. Most of these cells are highly active with strong capacity to enhance breast carcinogenesis through promoting cancer cells stemness and angiogenesis (5, 6). This CAF-related promotion of neo-vascularization is mediated through secretion of various pro-angiogenic factors such as SDF-1, VEGF-A and IL-8 (7–10). Neovascularization allows tumor growth and facilitates the dissemination of tumor cells, which are escorted and supported by CAFs all over the metastatic process from the primary site to the metastatic site (5, 9, 11, 12). Additionally, recent findings have highlighted the important role of CAFs in modulating the response to various types of therapies, and also their valuable role as prognostic tool (13–15). The prognostic function of CAFs depends on the variation in the expression of many important cancer-related genes. We have recently shown that the DNA methyl-transferase gene (DNMT1) is highly expressed in CAF cells as compared to their adjacent counterpart cells, and that DNMT1 upregulation activates breast stromal fibroblasts (16).

Furthermore, several studies have shown that the expression of DNMT1 is higher in various types of breast cancers compared with paired normal breast tissues, and that DNMT1 upregulation is associated with higher grades of the disease and poor survival (17–22). Therefore, it has become clear that CAF-related biomarkers have powerful prognostic value and can also guide the next-generation therapeutic approaches.

In the present report we have shown that DNMT1 controls the pro-angiogenic role of active breast stromal fibroblasts (BSFs) through positive regulation of two key angiogenic factors VEGF-A and IL-8. In addition, we provide clear information on the prognostic value of CAF-DNMT1 for LABC patients.

## Materials and Methods

### Cells and Cell Culture

Breast fibroblast cells (CAF-64 and TCF-64) were obtained and used as previously described (23). HUVEC cells were purchased from ATCC. Cells were regularly screened for mycoplasma contamination using MycoAlert Mycoplasma Detection Kits (Lonza). All supplements were obtained from Sigma (Saint Louis, MO, USA) except for antibiotic and antimycotic solutions, which were obtained from Gibco (Grand Island, NY, USA). Cells were maintained at 37°C in humidified incubator with 5% CO_2_.

### Cellular Lysate Preparation and Immunoblotting

This has been performed as previously described (24). Antibodies directed against DNMT1 (ab19905), IL-8 (ab52612) and VEGF-A (ab46154) were purchased from Abcam (Cambridge, MA), mTOR (7C10)/p.mTOR (Ser2448, D9C2), HIF-1α and Glyceraldehydes-3-phosphate dehydrogenase (GAPDH, FL-335) from Cell Signaling (Danvers, MA). The immunoblotting experiments were repeated at least 2 times.

### RNA Purification and qRT-PCR

Total RNA was purified using the miRNeasy mini kit (Qiagen, UK) according to the manufacturer’s instructions and was treated with RNase-free DNase. One (1) µg RNA was used to synthesize cDNA utilizing Advantage RT-PCR kit (Clontech Laboratories, Mountain View, CA, US). Quantitative RT-PCR was performed in triplicate using 4 µl cDNA mixed with 2x FastStart Essential DNA Green qPCR mastermix (Roche, New York, NY, US) and 0.3 µM forward and reverse primers. Amplifications were performed utilizing the LightCycler 96 Real-time PCR detection system (Roche) using the following cycle conditions: 95°C for 10 min (1 cycle); 95°C for 10 sec, 59°C for 20 sec, 72°C for 30 sec (45 cycles). GAPDH expression levels were used for normalization, and gene expression differences were calculated using the threshold cycle (Ct). Three independent experiments were performed for each reaction, and the obtained values were plotted as mean ± SD. The respective primers are:

*GAPDH*: 5’-GAGTCCACTGGCGTCTTC-3’ and 5’-GGGGTGCTAAGCAGTTGGT-3’

*VEGF-A:*5’- CCCACTGAGGAG TCCAACAT -3’ and 5’- TGGATGGTGGTACAGTCAGAG C -3’

*IL-8:* 5’- GAT CCACAAGTCCTTGTTCCA -3’ and 5’- GCT TCCACATGTCCTCACAA -3’

### SiRNA Transfection

The transfections using DNMT1-siRNA (Origene, SR301244A) and control–siRNA were carried out using the RNAi Fect reagent (Qiagen) as recommended by the manufacturer.

### Viral Infection

Lentivirus-based vector bearing *DNMT1*–ORF as well as its corresponding control (GeneCopoeia) were used to prepare the lentiviral supernatant from 293FT cells. Lentiviral supernatants were collected 48 h post-transfection, filtered and used for infection. 48 h later, media were replaced with complete media and cells were grown for 3 days.

### DNMT1-ORF Transfection

Lentivirus-based vectors bearing *DNMT1*-ORF, and the corresponding control were used to carry out transfection of BSF cells using human dermal fibroblast nucleofector 2000 transfection kit (Invitrogen) following the manufacturer’s recommendations. After 2 weeks, transfected cells were selected by puromycin (1µg/mL).

### ELISA Assays

Supernatants from 24 h fibroblast cell cultures were harvested, and ELISA was performed according to the manufacturer’s instructions (R&D Systems). The OD was used at 450-nm on a standard ELISA plate-reader. These experiments were performed in triplicates, and were repeated three times.

### Conditioned Media

Log phase cells (80% confluent) were cultured in medium without serum for 24 h, and then media were collected, briefly centrifuged and filtered, and then cells were counted. The resulting supernatants were used either immediately (taking into account the number of cells) or were frozen at -80 °C until needed.

### HUVEC Endothelial Tube Formation Assay

The formation of capillary-like structures was assessed in a 96-well plate coated with ice-cold growth factor-reduced Matrigel (in vitro angiogenesis assay, Millipore). After solidification of the matrix at 37°C, 1x104 HUVEC cells were seeded onto the polymerized matrix in the presence of 200 µl of conditioned medium. Formation of capillary-like structure was photographed after 5 h of incubation and their number was counted. The total tube area was obtained from five random microscopic fields and expressed as a mean of three different experiments.

### Patients and Archived Clinical Materials

Formalin-fixed paraffin-embedded tissues were obtained from the Pathology Department at KFSH&RC with institutional review board approval (RAC#2160005). The study cohort consisted of 100 locally advanced breast cancer patients who were diagnosed between 2006 and 2013, with a median fellow up time of 52. 6 months. Written informed consent was not required and a waiver was granted since samples were anonymized to the research team.

### Immunohistochemistry Staining on FFPE Tissues

Immunohistochemistry for DNMT1 done on formalin-fixed, paraffin-embedded tissue using anti-DNMT1 antibody from Abcam (Cambridge, MA) overnight at a dilution of 1:500 and were stained using automated staining platform (Ventana). Envision + polymer (ready to use; Dako) was used as a secondary antibody. Color was developed with 3,3′-diaminobenzidine (DAB) and instant hematoxylin (Shandon) was used for counterstaining. The DNMT1 level was evaluated and verified by two qualified pathologists, who scored both the proportion of positive cells as well as the intensity of DNMT1 expression in both cancer cells and their stromal fibroblasts.

For CD31, the number of CD31-positive vessels was counted in five different highest fields of microvessel density (40x objective lens and 10x ocular lens). CD31[P2B1] (ab24590) was purchased from Abcam.

### Quantification of Protein Expression Level

The protein signal intensity of each band was determined using ImageQuant TL software (GE Healthcare). Next, dividing the obtained value of each band by the value of the corresponding internal control allowed a correction of the loading differences. The fold change in the protein levels was determined by dividing the corrected values by that of the control.

### Statistical Analysis

Statistical analysis was performed by the software package SAS version 9.4 (SAS Institute Inc., Cary, NC, USA). Continuous variables were compared by Student’s t-test and *P* values of 0.05 and less were considered as statistically significant. Kaplan-Meyer method was used in survival tables and curves, and the different subgroups were compared by the log-rank test.

## Results

### DNMT1 Positively Regulates VEGF-A and IL-8 in Breast Stromal Fibroblasts

**Figure 1:**
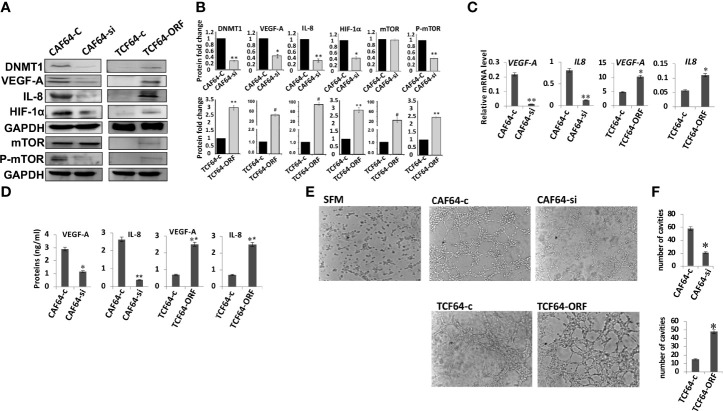
DNMTl controls the expression of VEGF-A and IL-8 in breast stromal fibroblasts and promotes their pro-angiogenic effects *in vitro*. CAF-64 cells were transfected with *DNMT1-siRNA* (CAF64-si) or with a plasmid bearing the *DNMT1* ORF (TCF64-orf). A scrambled sequence (CAF64-c) and an empty vector (TCF64-c) were used as controls, respectively. Whole-cell lysates were prepared, and then were used for immunoblotting analysis using specific antibodies against the indicated proteins. **(A)** Immunoblotting analysis **(B)** The histograms show the averaged protein level fold changes relative to the respective controls after normalization against the internal control GAPDH, while the level of the phospho-protein was further normalized to the level of the total protein. Error bars represent mean ± S.D (n=3). *P<0.05, **P<0.01 and ^#^P<0.0001. **(C)** Total RNA was extracted from the indicated cells and the mRNA levels of the indicated genes were assessed using qRT-PCR. Error bars represent mean ± S.D (n=3). *P<0.05 and **P<0.01. **(D)** SFCM from the indicated cells were collected after 24 h and the levels of the indicated proteins were determined by ELISA. Error bars indicate mean ± S.D (n=3). *P<0.05 and **P<0.01. **(E)** SFCM from the indicated cells were collected and used to treat HUVEC cells previously plated on matrigel (96-well plate), then incubated at 37°C for 8 hr. **(F)** Histograms show the average number of formed cavities. Error bars represent means ± S.D (n=3). *p<0.05.

We have recently shown that DNMT1 plays important role in the activation of breast stromal fibroblasts, which are known to promote angiogenesis in a paracrine manner (7, 16). Therefore, we sought to investigate the role of this methylation promoting gene in enhancing the pro-angiogenic effect of active fibroblasts. To this end, we have first investigated the implication of DNMT1 in regulating the expression of the pro-angiogenic factors VEGF-A and IL-8 within breast stromal fibroblasts (BSFs). To achieve this, DNMT1 was first ectopically expressed using a vector bearing DNMT1-ORF into TCF-64 cells (tumor counterpart fibroblasts present in a histologically normal part of the breast), while an empty vector was used as a control (TCF64-ORF and TCF64-c, respectively). **Figures 1A, B** show that DNMT1 upregulation increased the level of the VEGF-A and IL-8 proteins in TCF64-ORF cells compared to their corresponding control TCF64-c cells. This result was confirmed at the mRNA level for both genes (**Figure 1C**). Likewise, the secreted level of VEGF-A and IL-8 were also increased upon ectopic expression of DNMT1 in TCF-64 cells (**Figure 1D**). This indicates that DNMT1 positively controls the expression of both VEGF-A and IL-8. To confirm this, we decided to test the effect of DNMT1 down-regulation on the expression of both genes. Therefore, CAF-64 cells (cancer-associated fibroblasts present in the tumor) were transfected with either DNMT1 siRNA or a scrambled sequence that was used as a control, and then the levels of the VEGF-A and IL-8 proteins were assessed. **Figures 1A, B** show DNMT1 down-regulation accompanied by clear decrease in the protein level of VEGF-A and IL-8 in DNMT1-deficient cells compared to control cells. Similarly, the mRNA levels of both genes were significantly reduced in DNMT-1 deficient cells compared to controls (**Figure 1C**). Furthermore, the secreted levels of VEGF-A and IL-8 were also significantly reduced upon DNMT1 knockdown (**Figure 1D**). These results confirm the role of DNMT1 in the positive regulation of VEGF-A and IL-8, most likely at the mRNA level.

### DNMT1 Activates mTOR and Up-Regulates HIF-1α in Breast Stromal Fibroblasts

Next, we sought to delineate the molecular mechanism responsible for DNMT1-dependent regulation of VEGF-A and IL-8, which are both transcriptionally regulated by HIF-1α, a transcription factor at the heart of the angiogenic response (25, 26). Therefore, we checked the role of DNMT1 in controlling the expression of this transcription factor in breast stromal fibroblasts. **Figure 1A** shows that while the DNMT1 ectopic expression increased the level of HIF-1α in TCF-64 cells, DNMT1 down-regulation decreased the level of HIF-1α in CAF-64 cells, compared to the respective control cells. This suggests that DNMT1 positively regulates the expression of VEGF-A and IL-8 through positive regulation of their major transactivator gene HIF-1α. Since this gene is an important target of mTOR, we also checked the effect of DNMT1 on the expression of this protein kinase. Interestingly, DNMT1 upregulation in TCF-64 cells increased the level of the basal as well as the phosphorylated form of mTOR relative to control cells (**Figures 1A, B**). However, DNMT1 down-regulation reduced only the active form of the protein (P-mTOR) with only slight effect on the basal level of the protein (**Figures 1A, B**). This indicates that DNMT1 can activate mTOR and its downstream proangiogenic effector HIF-1α in breast fibroblasts.

### DNMT1 Promotes the Paracrine Pro-Angiogenic Effect of Active CAFs *In Vitro*

The fact that DNMT1 enhances the expression and the secretion of 2 major angiogenesis factors, prompted us to investigate the possible involvement of DNMT1 in promoting the paracrine pro-angiogenic effect of active breast fibroblasts. To this end, serum-free conditioned media (SFCM) collected from TCF64-ORF/TCF64-c and CAF64-si/CAF64-c cells were added separately to 96-well plates seeded with 2 × 10^4^ human umbilical vein endothelial cells (HUVECs) in matrigel and used for *in vitro* angiogenic assay, serum-free medium (SFM) was also added as negative control. After 5 h of incubation, the number of closed cavity constructions was significantly lower in the presence of SFCM from CAF64-si cells compared to SFCM from CAF64-c cells (**Figure 1E**). However, SFCM from TCF64-ORF significantly increased the number of meshs compared to SFCM from TCF64-c or SFM (**Figures 1E, F**). These results demonstrate the role of the stromal fibroblast DNMT1 protein in stimulating endothelial cell differentiation into capillary-like structures through a paracrine effect.

### Ectopic Expression of DNMT1 Enhances the Paracrine Pro-Angiogenic Effects of Breast Stromal Fibroblasts *In Vivo*

**Figure 2:**
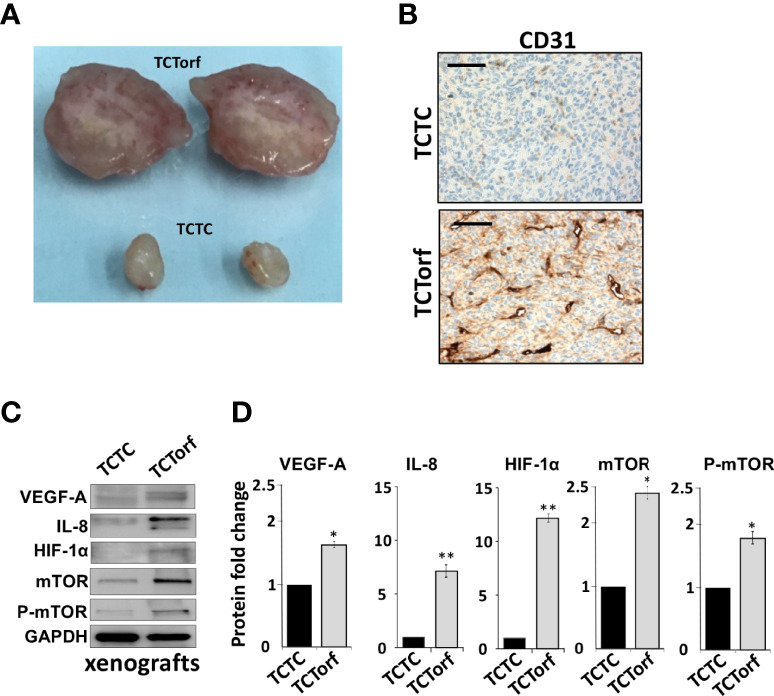
Ectopic expression of DNMT1 enhances the paracrine pro-angiogenic effects of breast stromal fibroblasts *in vivo*. Orthotopic BC xenografts were created by co-injecting MDA-MB-231 cells with TCF64-orf or TCF64-c cells under the nipple of nude mice as previously described (16). **(A)** Picture of excised tumors **(B)** Immunohistochemical staining was carried out on FFPE sections using an anti–CD-31 antibody. Scale bars represent 200 μM. **(C)** Whole-cell lysates were prepared from the excised tumors, and then were used for immunoblotting analysis using specific antibodies against the indicated proteins. **(D)** The histograms show the averaged protein level fold changes relative to the control (TCTC) after normalization against the internal control GAPDH, while the level of the phospho-protein was further normalized to the level of the total protein. Error bars represent mean ± S.D (n=3). *P<0.05 and **P<0.01.

To study the paracrine effect of DNMT1-expressing BSFs on vascular formation *in vivo*, we made use of previously created orthotopic BC xenografts by co-injecting MDA-MB-231 cells with TCF64-ORF (TCTorf) or TCF64-c (TCTC) cells in nude mice (16). Tumors were excised and a significant difference in tumor size and microvascular density were observed between tumors bearing TCF64-ORF and their controls (**Figure 2A**). To further confirm this, the level of CD31, an endothelial cell marker, was assessed in the orthotopic tumor xenografts by immunohistochemistry using anti-CD31 antibody. **Figure 2B** shows a higher density of CD31^+^ cells in tumors containing TCF64-ORF cells compared to tumors mixed with TCF64-c cells. To explore the molecular mechanism(s) that underlies the proangiogenic effect of TCF64-ORF cells, we assessed the level of the proangiogenic proteins in the two tumors by immunoblotting. **Figures 2C, D** show that the levels of VEGF-A, IL-8 as well as their upstream regulators mTOR and HIF-1α, were increased in TCTorf compared to TCTC tumors. These results show the role of DNMT1 in promoting the paracrine proangiogenic effect of breast stromal fibroblasts *in vivo*.

### Eugenol and Decitabine Suppress the Proangiogenic Effects of Breast Myofibroblasts by Inhibiting DNMT1 Expression

**Figure 3:**
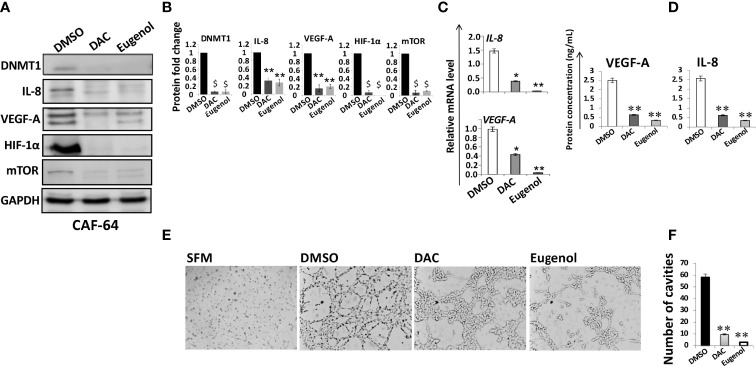
Eugenol and DAC suppress the proangiogenic effects of breast myofibroblasts by inhibiting DNMT1 expression. **(A)** CAF-64 cells were treated with the indicated concentrations of DAC and eugenol while DMSO was used as control, and then whole-cell lysates were prepared and utilized for immunoblotting analysis using specific antibodies against the indicated proteins. The numbers under the bands represent fold changes relative to the control (DMSO) after normalization against the internal control GAPDH. **(B)** The histograms show the averaged protein level fold changes relative to the control after normalization against the internal control GAPDH. Error bars represent mean ± S.D (n=3). ** P<0.01 and ^$^P<0.001. **(C)** Total RNA was extracted from CAF-64 cells treated as shown, and the mRNA levels of the indicated genes were assessed using qRT–PCR. Error bars represent mean± S.D (n=3).*P ≤ 0.05, **P ≤ 0.01 **(D)** CAF-64 cells were treated as indicated in SFM for 24 h, and then SFCM were collected, and the levels of the indicated proteins were determined by ELISA. Error bars indicate mean ± S.D (n=3). **P ≤ 0.01. **(E)** HUVEC cells previously plated on matrigel (96-well plate) were treated with SFCM collected as described in **(D)**, while SFM was used as negative control, and then cells were incubated at 37°C for 8 hr. **(F)** Histogram shows the average number of formed cavities. Error bars represent means ± S.D (n=3). **P ≤ 0.01.

We have recently shown that the DNMT inhibitor decitabine (5-Aza-2’-deoxycytidine, DAC) and eugenol suppress the pro-carcinogenic effects of active fibroblasts through targeting DNMT1 (27). Therefore, we decided to investigate the role of these two DNMT1 inhibitors on the proangiogenic process of active fibroblasts. To this end, CAF-64 cells were treated for 24 h with eugenol (1µM) or DAC (5µM), while DMSO was utilized as a negative control, and then whole cell lysates were prepared. The immunoblotting analysis showed that DNMT1 protein level declined along with reduction in the protein level of VEGF-A and IL-8 in cells treated with DAC or eugenol as compared to controls (**Figures 3A, B**). Similarly, DAC and eugenol significantly reduced the mRNA levels of both VEGF-A and IL-8 (**Figure 3C**). Since DNMT1 controls the expression of HIF-1α and mTOR, we decided to check the effect of DNMT1 inhibition on such angiogenesis regulatory factors. Indeed, eugenol and DAC strongly inhibited the expression of both mTOR/P-mTOR and HIF1α (**Figures 3A, B**). This indicates that eugenol and DAC are strong suppressors of the major angiogenesis effectors and regulators, probably through controlling DNMT1.

To confirm this, we have shown that eugenol and DAC significantly reduced more than 5 fold the levels of secreted VEGF-A and IL-8 from CAF cells as compared to control cells (**Figure 3D**). This shows that eugenol and DAC could suppress the paracrine proangiogenic effect of CAF cells through DNMT1 inhibition. To verify this, we investigated the effect of eugenol- and DAC -treated CAF cells on the differentiation of endothelial cells *in vitro*. Therefore, CAF-64 cells were either DMSO-treated or challenged with either eugenol (1µM) or DAC (5µM) in SFM for 24 h to generate SFCM that have been used to treat HUVEC cells (0.5×10^5^) on matrigel pre-coated 96-well plate for 16 h at 37°C. **Figures 3E, F** show that while HUVEC cells were differentiated into mech structures in the presence of DMSO-SFCM, only marginal differentiation occurred in the presence of eugenol- and DAC-SFCM. Together, these results indicate that eugenol and decitabine suppress the paracrine pro-angiogenic effect of CAF cells through inhibiting DNMT1 and its downstream angiogenic effectors VEGF-A and IL-8.

### Correlation of DNMT1 Expression in Cancer and Stromal Fibroblasts With Clinicopathological Parameters

Next, we sought to investigate the predictive value of DNMT1 expression levels in cancer cells as well as their stromal fibroblasts as a candidate biomarker for clinical outcome of patients with LABC. Remarkably, 69% of the patients were less than 50 years old and 60% of patients had tumor sizes more than 5 cm (**Table 1**). Thirty-three (33) patients developed recurrence and 13 died (**Table 1**). Furthermore, 70% of the patients had high tumor stage, while 48% of the tumors were of grade 3 (**Table 1**). Estrogen receptor positive (ER+)/Her2+ patients represented 26%, ER+/Her2- represented 31%, ER-/Her2+ represented 25%, while ER-/Her2- represented 18% (**Table 1**).

**Table 1:** Correlations between DNMT1 expression and clinicopathological features in breast cancer patients.

Parameter	Total n=100 (%)	DNMT1 in cancer cells		*P* value
		>10%	≤10%	
**Age** ≤50 >50	69 (69.00) 31 (31.00)	35 (35.00) 14 (14.00)	34 (34.00) 17 (17.00)	0.6068
**Survival status** Alive Died	87 (87.00) 13 (13.00)	39 (39.00) 12 (12.00)	48 (48.00) 1 (1.00)	**0.0014**
**HIS-Subtype** Invasive Ductal Ca 1,4 Invasive Ductal Ca with DCIS Infiltrating Lobular Ca Other	83 (83.00) 3 (3.00) 11 (11.00 2 (2.00) 1 (1.00)	39 (39.00) 1 (1.00) 7 (7.00) 2 (2.00) 0 (0.00)	44 (44.00) 2 (2.00) 4 (4.00) 0 (0.00) 1 (1.00)	0.3712
**Tumor stage ** T2 T3 T4 Tx	29 (29.00) 31 (31.00) 39 (39.00) 1 (1.00)	14 (14.00) 14 (14.00) 20 (20.00) 1 (1.00)	15 (15.00) 17 (17.00) 19 (19.00) 0 (0.00)	0.8909
**Grade** G1 G2 G3 Gx	2 (2.00) 48 (48.00) 48 (48.00) 2 (2.00)	2 (2.00) 26 (26.00) 19 (19.00) 2 (2.00)	0 (0.00) 22 (22.00) 29 (29.00) 0 (0.00)	0.0676
**Recurrence** No Yes	67 (67.00) 33 (33.00)	34 (34.00) 15 (15.00)	33 (33.00) 18 (18.00)	0.6187
**Progression** No Yes	17 (17.00) 83 (83.00)	9 (9.00) 40 (40.00)	8 (8.00) 43 (43.00)	0.7212
**Tumor size** ≤2cm 2-5cm > 5cm	1 (1.16) 33 (38.37) 52 (60.47)	0 (0.00) 17 (19.77) 27 (31.40)	1 (1.16) 16 (18.60) 25 (29.07)	0.9108
**Parameter**	**Total n=100** **(%)**	**DNMT1 in fibroblasts**		***P* value**
		**>10%**	**≤10%**	
**Age** ≤50 >50	69 (69.00) 31 (31.00)	44 (44.00) 24 (24.00)	25 (25.00) 7 (7.00)	0.1759
**Survival status** Alive Died	87 (87.00) 13 (13.00)	25 (25.00) 10 (10.00)	62(62.00) 3 (3.00)	**0.01081**
**HIS-Subtype** Invasive Ductal Ca 1,4 Invasive Ductal Ca with DCIS Infiltrating Lobular Ca Other	83 (83.00) 3 (3.00) 11 (11.00) 2 (2.00) 1 (1.00)	55 (55.00) 1 (1.00) 9 (9.00) 2 (2.00) 1 (1.00)	28 (28.00) 2 (2.00) 2 (2.00) 0 (0.00) 0 (0.00)	0.4853
**Tumor stage ** T2 T3 T4 Tx	29 (29.00) 31 (31.00) 39 (39.00) 1 (1.00)	22 (22.00) 21 (21.00) 20 (20.00) 1 (1.00)	7 (7.00) 10 (10.00) 19 (19.00) 0 (0.00)	0.8909
**Grade** G1 G2 G3 Gx	2 (2.00) 48 (48.00) 48 (48.00) 2 (2.00)	2 (2.00) 26 (26.00) 19 (19.00) 2 (2.00)	0 (0.00) 22 (22.00) 29 (29.00) 0 (0.00)	0.0676
**Recurrence** No Yes	67 (67.00) 33 (33.00)	34 (34.00) 15 (15.00)	33 (33.00) 18 (18.00)	0.6187
**Progression** No Yes	17 (17.00) 83 (83.00)	9 (9.00) 40 (40.00)	8 (8.00) 43 (43.00)	0.7212
**Tumor size** ≤ 2cm 2-5cm > 5cm	1 (1.16) 33 (38.37) 52 (60.47)	0 (0.00) 17 (19.77) 27 (31.40)	1 (1.16) 16 (18.60) 25 (29.07)	0.9108

**Figure 4:**
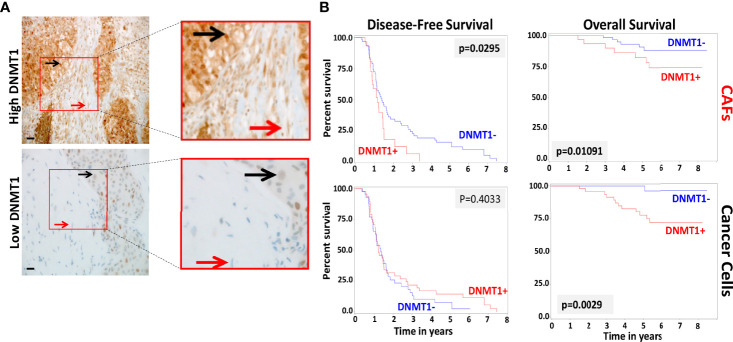
Loss of DNMT1 in stromal and tumoral cells is predictive of poor disease-free and overall survival. **(A)** Tissue sections cut from formalin-fixed paraffin embedded breast tumors were immunostained with an anti-DNMT1 antibody. Red arrows indicate stromal fibroblasts, black arrows indicate cancer cells. (Envision 60x). **(B)** Kaplan-Meier analysis of overall survival (OS) and disease-free survival (DFS).

In order to study the role of DNMT1 in the prognosis of LABC patients, a total of 100 breast pretreatment tumor tissues were assessed for the expression of the DNMT1 protein in cancer cells and their adjacent stromal fibroblasts. **Figure 4A** shows the immunostaining of DNMT1 in both tumor as well as stromal cells from breast cancer tissues. The level of DNMT1 immunostaining in both cancer and stromal cells was classified into 2 subgroups: low (≤10% DNMT1-positive cells) and high (>10% DNMT1-positive cells). DNMT1 expression was low in 51% epithelial and 32% fibroblast cells, while DNMT1 was highly expressed in 49% epithelial and 68% fibroblast cells (**Table 2**). The level of DNMT1 in fibroblasts in the different breast cancer subtypes showed association with ER/Her2 status. Indeed, significant correlation (*P* = 0.0121) was observed between low DNMT1 level in fibroblasts and lack of ER/PR (**Table 2**). However, no correlation was observed between DNMT1 level in cancer cells and ER/Her2 expression levels (**Table 2**). Interestingly, the level of DNMT1 was highly correlated with patient survival in both breast cancer cells (*P*=0.0014) and fibroblasts (*P*=0.01081) (**Table 2**). Indeed, high DNMT1 expression levels were highly correlated with poor patient survival (**Table 2**). However, no significant correlation was observed between the level of DNMT1 in tumor or stromal cells and the other clinicopathological parameters (**Table 2**).

**Table 2:** Expression of DNMT1 in cancer cells and stromal fibroblasts by breast cancer ER/Her2 subtypes.

Cancer cells		Total	ER(+ve)/Her2(+ve)	ER(+ve)/Her2(-ve)	ER(-ve)/Her2(+ve)	ER(-ve)/Her2(-ve)	*P value*
		n=100 (%)					
**DNMT1**	>10%	49 (49.00)	12 (12.00)	20 (20.00)	9 (9.00)	8 (8.00)	0.1784
	≤10%	51 (51.00)	14 (14.00)	11 (11.00)	16 (16.00)	10(10.00)
**Fibroblasts**		Total	ER(+ve)/Her2(+ve)	ER(+ve)/Her2(-ve)	ER(-ve)/Her2(+ve)	ER(-ve)/Her2(-ve)	***P value***
		n=100 (%)			
**DNMT1**	>10%	68 (68.00)	16 (16.00)	28 (28.00)	13 (13.00)	11 (11.00)	0.0121
	≤10%	32 (32.00)	10 (10.00)	3 (3.00)	12 (12.00)	7 (7.00)

### DNMT1 Expression in Both Breast Cancer Cells and Their Stromal Fibroblasts Predicts Survival

Kaplan-Meier plots indicate significant association between DNMT1 expression levels in cancer cells and their stromal fibroblasts and patient’s overall survival (OS) (*P* = 0.0029 and *P* = 0.01091, respectively) (**Figure 4B**). Indeed, patients with tumors expressing high level of DNMT1 in stromal fibroblasts or cancer cells had significantly poorer OS rates (**Figure 4B**). In contrast, low DNMT1 levels were significantly associated with better OS (**Figure 4B**). However, while high DNMT1 levels in fibroblasts correlated significantly with poorer disease-free survival (DFS) (P = 0.0295), DNMT1 expression level in cancer cells did not significantly correlate with patient DFS (*P* = 0.4033) (**Figure 4B**).

The poor outcome of patients with high DNMT1 expression in stromal fibroblasts (*P* = 0.01204) and cancer cells (*P* = 0.0187) was confirmed by univariate Cox regression analysis (**Table 3**). Furthermore, multivariate Cox regression analysis was conducted to investigate the link between the prognostic power of DNMT1 expression level and other well-known breast cancer risk factors. **Table 4** shows that the DNMT1 level in cancer cells and their stromal fibroblasts is a strong independent predictor of OS (*P* = 0.011) and (*P* = 0.0360), respectively. Furthermore, DNMT1 level in fibroblasts was also an independent prognostic factor for DFS (*P* = 0.0492) (**Table 4**).

**Table 3:** Univariate Cox proportional regression analysis on 5-year overall and disease-free survival of 100 LABC patients.

Parameter	Overall survival			Disease-free survival		
	Hazard Ratio	95% Hazard Ratio Confidence Limits	*P value*	Hazard Ratio	95% Hazard Ratio Confidence Limits	*P value*
**DNMT1 (Fibroblasts)** **≤10%** **>10%**	1 0.421	0.141-1.255	0.01204	1 0.581	0.353-0.955	**0.0322**
**DNMT1 (Cancer cells)** **≤10%** **>10%**	1 0.087	0.011-0.666	**0.0187**	1 1.207	0.635-0.997	0.4068
**Clinical_Tumor_Stage** T2 T3, T4, Tx	1 1.212	0.834-1.762	0.3139	1 0.796	0.635-0.997	0.0469
**Grade** G1, G2 G3, Gx	1 1.011	0.577-1.771	0.9686	1 0.972	0.817-1.155	0.7446
**Tumor size** ≤ 5cm > 5cm	1 2.743	0.754-9.972	0.1255	1 0.980	0.615-1.559	0.9308
**AGE** ≤50 >50	1 1.003	0.952-1.058	0.8979	1 1.002	0.980-1.025	0.8626

**Table 4:** Multivariate Cox Regression analysis on 5-year overall and disease-free survival.

Parameter	OS			DFS		
	Hazard Ratio	95% Hazard Ratio Confidence Limits	*P value*	Hazard Ratio	95% Hazard Ratio Confidence Limits	*P value*
**DNMT1 (Cancer cells)**	0.014	0.000-0.375	**0.011**	1.385	0.830-2.312	0.212
**ER-PR-Status** ER(+ve)/PR(-ve) ER(-ve)/PR(-ve)	2.559 0.935	0.277-23.675 0.109-8.054	0.4079 0.9512	1.001 1.001	0.354-2.830 0.347-1.789	0.9982 0.5694
**Tumor-Stage**	10.207	1.830-56.943	0.0081	0.953	0.626-1.450	0.8213
**Grade**	0.048	0.006-0.378	0.0040	1.048	0.764-1.437	0.7701
**Tumor size**	2.576	0.341-19.482	0.3594	0.893	0.464-1.717	0.7338
**HIS-Subtype** ER(+ve)/Her2(-ve) ER(-ve)/Her2(+ve) ER(-ve)/Her2(-ve)	0.862 7.318 52.729	0.074-10. 045 0.716-74.791 2.055-1353.196	0.905 0.093 0.016	0.535 1.328 0.800	0.254-1.129 0.362-2.031 0.367-1.898	0.1007 0.7261 0.6657
**AGE**	0.871	0.764-0.993	0.0392	0.999	0.966-1.034	0.9507
**DNMT1 (Fibroblasts)**	0.661	0.119-3.673	0.0360	0.426	0.308-0.998	0.0492
**ER-PR-Status** ER(+ve)/PR(-ve) ER(-ve)/PR(-ve)	1.388 0.552	1.167-11.522 0.064-4.753	0.7612 0.5886	0.852 0.9	0.291-2.497 0.385-2.104	0.7706 0.8084
**Tumor-Stage**	7.898	1.606-38.836	0.0110	0.936	0.608-1.442	0.7642
**Grade**	0.163	0.018-1.461	0.1049	0.998	0.709-1.407	0.9929
**Tumor size**	1.598	0.242-10.555	0.6268	0.765	0.394-1.484	0.4276
**HIS-Subtype** ER(+ve)/Her2(-ve) ER(-ve)/Her2(+ve) ER(-ve)/Her2(-ve)	0.563 12.537 34.608	0.045-7.012 1.715-91.654 2.105-569.059	0.6551 0.0127 0.0131	0.574 0.711 0.778	0.272-1.208 0.293-1.724 0.324-1.834	0.1436 0.4506 0.5557
**AGE**	0.886	0.789-0.995	0.0416	1.005	0.970-1.040	0.7893

## Discussion

When active, CAFs boost breast carcinogenesis through promoting several pro-carcinogenic processes such as angiogenesis (5, 6). Our recent findings indicate that DNMT1 is highly expressed in CAFs and that when upregulated it enables the activation of breast stromal fibroblasts (16). The present findings indicate that DNMT1 controls the paracrine pro-angiogenic potential of BSFs through positive regulation of both pro-angiogenic factors VEGF-A and IL-8. Indeed, while DNMT1 knock-down reduced the expression level of both genes and consequently the pro-angiogenic capacity of CAFs, ectopic expression of DNMT1 up-regulated VEGF-A and IL-8 and enhanced the pro-angiogenic ability of BSFs both *in vitro* and *in vivo*. In a previous study, Achour et al., have shown that DNMT1 forms a heterocomplex with ICBP90, which positively controls the expression of VEGF (28). To elucidate the molecular pathway underlying the DNMT1-dependent induction of the VEGF-A and IL-8 genes, we have shown that DNMT1 is an activator of HIF-1α and mTOR two upstream activators of VEGF-A (29). Since DNMT1 positively regulates the expression of these genes, DNMT1 may not directly control their expression through methylation, which is a gene repressor process. In fact, approximately 30% of the upregulated genes in DNMT1 knockout cells do not contain dense CpG islands (30), confirming DNMT1-dependent gene expression regulation in a methylation-independent manner. This suggests that DNMT1 may indirectly regulate these genes through controlling their upstream transactivators. Indeed, we have recently shown that DNMT1 positively controls the expression of AUF1, which is part of the IL-6/STAT3/NF-κB positive feedback loop in breast fibroblasts (16, 31). This indicates that DNMT1 is involved in the regulation of a plethora of genes involved in various physiological processes including angiogenesis.

In recent years natural products showed promising ability to repress the procarcinogenic effects of active CAFs through multiple mechanisms. Thereby, agents that can normalize CAFs may improve the efficiency of traditional tumor cell-directed therapy. Consequently, we tested the effect of the nontoxic and pharmacologically safe dietary compound eugenol, and we compared its effects with the well-known DNMTs inhibitor decitabine, on the expression of HIF-1α/mTOR as well as VEGF-A and IL-8 and the related pro-angiogenic effect of active BSFs. We have shown that both eugenol and DAC repress VEGF-A and IL-8 expression and secretion as well as the expression and activation of HIF-1α/mTOR in CAFs, and consequently suppress their pro-angiogenic paracrine effect *in vivo* and *in vitro*. Several studies reveal the therapeutic potential of eugenol in cancer prevention and treatment. Indeed, administration of eugenol inhibits angiogenesis as evidenced by changes in the activities of VEGF-A and VEGFR1 in a rat model of gastric carcinogenesis (32). Additionally, a recent study reported that eugenol promotes cisplatin cytotoxicity against TNBC by inhibiting the NF-*κ*B signaling pathway (33). Notably, the present report shows that eugenol possesses anti-angiogenic effect indirectly through inhibition of the pro-angiogenic effect of CAFs. This suggests that eugenol could be of great value for cancer prevention and/or treatment by preventing the pro-vascularization potential of active stromal myofibroblasts.

Additionally, these findings suggest that high levels of DNMT1 could be associated with poor patient survival. Thereby, we investigated the link between the level of DNMT1 in breast cancer cells and their stromal fibroblasts with the survival of patients with LABC treated with neoadjuvant chemotherapy ± Trastuzumab. We have found significant association between high tumor expression of DNMT1 and shorter OS of LABC patients. Similar association has been previously reported in breast cancer (34), malignant lymphoma (35), renal cell carcinoma (36), bladder cancer (37) and pancreatic cancer (38). Furthermore, we have shown here that high level of DNMT1 in stromal fibroblasts is also associated with significantly shorter OS and DFS of LABC patients. This shows that the importance of DNMT1 as prognostic biomarker for LABC patients may not be restricted to malignant cells but also their stromal adjacent fibroblasts. This suggests that high DNMT1 level in breast malignant cells or their adjacent CAFs can significantly predict high risk or recurrence. This points to DNMT1 as promising therapeutic target in the neoadjuvant treatment of LABC patients. In fact, this possibility has been explored for several tumors (39–42).

## Conclusions

The present findings show that DNMT1 positively controls two major angiogenesis factors VEGF-A and IL-8 in breast stromal fibroblasts, and consequently DNMT1 upregulation in these cells promotes angiogenesis in a paracrine manner. Interestingly, these DNMT1-related pro-angiogenic effects can be suppressed by 2 DNMT1 inhibitors decitabine and eugenol. Furthermore, we present clear indication that high levels of DNMT1 in tumor cells as well as their adjacent stromal fibroblasts predict poor survival post-neoadjuvant treatment of locally advanced breast cancer patients. Thereby, DNMT1 level in both cancer cells and their adjacent stromal fibroblasts could constitute a powerful prognostic biomarker for these high-risk patients who need downstaging tumors to facilitate breast conservation therapy.

## Data Availability Statement

The original contributions presented in the study are included in the article/supplementary material. Further inquiries can be directed to the corresponding author.

## Ethics Statement

Written informed consent was not required and a waiver was granted since the study was retrospective and samples were anonymized to the research team. This was granted by the institutional review board approval (RAC#2160005).

## Author Contributions

All authors contributed to the article and approved the submitted version. Conception and design: AA; Development of methodology and acquisition of data: LA-K, AE, AT, and MA; Analysis and interpretation of data: LA-K, TA-T, and AA; Writing of the manuscript: LA-K, and AA.

## Funding

This work was solely supported by the King Faisal Specialist Hospital and Research Centre.

## Conflict of Interest

The authors declare that the research was conducted in the absence of any commercial or financial relationships that could be construed as a potential conflict of interest.

## Publisher’s Note

All claims expressed in this article are solely those of the authors and do not necessarily represent those of their affiliated organizations, or those of the publisher, the editors and the reviewers. Any product that may be evaluated in this article, or claim that may be made by its manufacturer, is not guaranteed or endorsed by the publisher.
